# Factors Affecting Long-Term Compliance with Rigid Gas-Permeable Contact Lens Wear in Patients with Keratoconus

**DOI:** 10.3390/jcm11041091

**Published:** 2022-02-18

**Authors:** Yu Xue, Jiaqi Zhou, Zhi Chen, Feng Xue, Li Zeng, Xiaomei Qu, Xingtao Zhou

**Affiliations:** 1 Eye Institute and Department of Ophthalmology, Eye & ENT Hospital, Fudan University, Shanghai 200031, China; yvonnehsueh@163.com (Y.X.); qiqi_zjq@163.com (J.Z.); peter459@aliyun.com (Z.C.); lilyxue80@vip.163.com (F.X.); doctzengli@163.com (L.Z.); quxiaomei2002@126.com (X.Q.); 2 NHC Key Laboratory of Myopia (Fudan University), Key Laboratory of Myopia, Chinese Academy of Medical Sciences, Shanghai 200031, China; 3 Shanghai Research Centre of Ophthalmology and Optometry, Shanghai 200031, China; 4 Shanghai Engineering Research Centre of Laser and Autostereoscopic 3D for Vision Care (20DZ2255000), Shanghai 200031, China

**Keywords:** keratoconus, rigid gas-permeable contact lens, compliance, questionnaire, corneal cross-linking

## Abstract

The purpose of the study was to investigate the factors affecting long-term compliance with rigid gas-permeable contact lens (RGPCL) wear in patients with keratoconus (KC). A total of 189 patients with KC (374 eyes) were included in the study, and were divided into two groups: the compliant group and the non-compliant group. Corneal topographic measurements, refractive results, and RGPCL parameters were compared between the two groups. A vision-related quality of life questionnaire was completed by all of the patients. The results demonstrated that patients diagnosed with bilateral KC were more compliant with RGPCL wear than patients diagnosed with unilateral KC (*p* = 0.0167). There were no significant differences between the compliant and non-compliant groups in terms of their corneal topographic measurements, refractive results, RGPCL parameters, or corneal cross-linking surgery history (all *p* > 0.05). In contrast, KC patients’ subjective experience with RGPCL wear—including visual acuity (*p* = 0.006), overall satisfaction (*p* < 0.001), quality of life (*p* < 0.001), and good adaptation during the short-term (*p* < 0.001)—had a significant effect on the long-term compliance with RGPCL wear. In conclusion, patients’ subjective experiences, rather than their ocular biometrics, significantly influence their long-term compliance with RGPCL wear.

## 1. Introduction

Keratoconus (KC) is a bilateral, noninflammatory corneal ectasia characterised by progressive corneal thinning and protrusion, leading to increasing myopia, irregular astigmatism, and an eventual scarring of the cornea, with poor-quality vision [1,2]. The prevalence of KC was reported to be 1.38 per 1000 in the general population [3].

Depending on the severity of the disease, different options for KC management are available. Irregular astigmatism in the early stages of KC can be corrected with spectacles; as the disease progresses, spectacles can no longer meet the needs of visual rehabilitation. Rigid gas-permeable contact lenses (RGPCLs) are most commonly used for the non-surgical management of patients with KC. RGPCLs provide better visual acuity and quality of life than spectacles [4,5]. Moreover, in a patient with higher keratometry or lower thinnest corneal thickness who has failed a trial of RGPCLs, the alternative contact lens options include hybrid lenses, piggy-back lenses, and scleral lenses.

RGPCLs create a thin lacrimal lens between the irregular corneal surface and the smooth posterior regular surface of the contact lens, which can neutralise most of the corneal astigmatism error [6]. In addition, studies have shown that the use of RGPCLs can delay the need for surgery in most patients with KC [7,8].

Surgical management options are available for KC, including corneal collagen cross-linking (CXL), intra-corneal ring segments, and corneal grafting [9]. CXL surgery, which was first introduced by Wollensak et al. [10], can halt or slow down the progression of KC. In CXL surgery, riboflavin (vitamin B2) is administered in conjunction with ultraviolet A (UVA, 370 nm). The interaction of riboflavin and UVA leads to the formation of reactive oxygen species, which induce covalent bonds between collagen fibrils in the corneal stroma, with the consequent biomechanical stiffening of the cornea [10]. Patients who are not satisfied with their visual acuity with spectacles after CXL surgery could be recommended for a trial of RGPCL wear.

Although RGPCLs can improve corrected distance visual acuity (CDVA), patients may stop wearing RGPCLs due to discomfort, inconvenience, and intolerance. A recent study found that CXL could increase RGPCL tolerance due to decreased corneal sensitivity and corneal flattening after CXL [11].

There are studies discussing the effect of long-term RGPCL wear on KC progression [4] and vision-related quality of life with RGPCL wear in patients with KC [12,13,14], while there are few studies assessing the compliance of patients with KC wearing RGPCLs.

This study aimed to determine the factors affecting long-term compliance with RGPCL wear in patients with KC, and to provide clinical recommendations for better KC management. The factors were mainly recorded from clinical data and patients’ answers to the questionnaire, and were categorized into objective and subjective factors for further analysis and discussion. Objective factors included the patients’ clinical data, such as corneal topographic measurements, refractive results, RGPCL parameters, and CXL surgery history. Subjective factors included the patients’ experience with RGPCL wear, such as visual acuity, overall satisfaction, quality of life, and good adaptation in the short-term.

## 2. Materials and Methods

### 2.1. Study Subjects

A total of 374 eyes of 189 patients with KC, who were fitted with an RGPCL at the Eye & ENT Hospital, Fudan University, between February 2009 and June 2019, were included and divided into two groups: the compliant group (patients with KC who were still wearing RGPCLs) and the non-compliant group (patients with KC who stopped wearing RGPCLs). The inclusion criteria of clinical KC were as follows: an asymmetric bowtie pattern with or without skewed axes revealed by corneal topography, and at least one KC clinical sign detected by slit-lamp examination (localised stromal thinning, Vogt’s striae, Fleischer’s ring, conical protrusion, or an anterior stromal scar). Unilateral KC was considered to be present if a patient had KC in one eye but did not meet the diagnostic criteria in the contralateral eye. This study was approved by the Ethics Committee of Eye & ENT Hospital, Fudan University, and adhered to the tenets of the Declaration of Helsinki.

### 2.2. Refraction

The data included CDVA with RGPCL wear and the final specifications of the best fit (base curve, power, and total diameter). In addition, in order to compare the visual acuity between RGPCLs and spectacles, CDVA with spectacles was measured. The spherical equivalent and cylinder with spectacles were also recorded. CDVA was converted to the logarithm of the minimum angle resolution, and was used for further analysis.

### 2.3. Corneal Topography

A Pentacam HR imaging system (Oculus, Wetzlar, Germany), which uses the Scheimpflug imaging technique, was used to perform the corneal topographic examinations. The rotating camera was set to capture 25 Scheimpflug slit images in approximately 2 s. All of the procedures were performed by experienced operators, and all of the patients were instructed to blink once before the image acquisition. In order to avoid miscalculations of poor imaging quality, the measurement quality was displayed in a specific Quality Specification (QS) window. Only results with ‘OK’ in the QS window, indicating good image quality, were included in the statistical analysis. Each eye was examined three times to obtain the mean value.

The following parameters were evaluated in this study: the thinnest corneal thickness (TCT), flat keratometry (K1), steep keratometry (K2), and maximum keratometry (Kmax).

### 2.4. Contact Lens Fitting

The fitting procedure of the RGPCLs was performed based on the standard ‘three-point touch’ method. The lens fit was assessed using a slit-lamp biomicroscope. The fit was deemed good if there was good centration, adequate movement, and a ‘three-point touch’ fluorescein pattern. Rose-K or aspheric RGPCL designs were used (Hiclear HLK, Brighten Optix Co., Taipei, Taiwan, China; Freshkon, Oculus Pvt. Ltd., Shanghai, China; Menicon Z, Menicon Co. Ltd., Nagoya, Japan).

### 2.5. Collagen Cross-Linking Procedure

The patients were placed in a supine position, and anaesthetic eye drops were applied preoperatively, after which a lid speculum was used. The corneal epithelium remained intact. Paracel (Avedro, Waltham, MA, USA, containing 0.25% riboflavin-5-phosphate) in corneal epithelial trephine (Model 52503B; 66 Vision-Tech, Suzhou, China) was used to completely cover the cornea for 4 min. VibeX Xtra (Avedro, Waltham, MA, USA, containing 0.25% riboflavin-5-phosphate) was then used to rinse and cover the cornea with corneal epithelial trephine for 6 min. A KXL System (Avedro) was used to conduct ultraviolet treatment with pulsed illumination for 1 s at 45 mW/cm^2^, delivering a surface dose of 7.2 J/cm^2^. This treatment step lasted for 5 min and 20 s. Subsequently, a bandage contact lens was applied. Antibiotic drops were applied for 1 week, and topical steroids were administered for 16 days (four times a day initially, then reduced to once every 4 days). The bandage contact lens was removed on postoperative days 1–5, according to epithelialisation. RGPCLs were prescribed 1 month after CXL.

The indications for CXL surgery were as follows: diagnosis with progressive KC with a thinnest corneal thickness (TCT) greater than or equal to 400 μm, or patients younger than 40 years. KC progression was confirmed by an increase in maximum keratometry (Kmax) of at least 1.00 diopter (D) in the past year.

Patients wearing RGPCLs discontinued their use for at least two weeks before surgery.

### 2.6. Questionnaire Survey

The demographic and vision-related quality of life questionnaire was prepared in English and then translated into Chinese. All of the subjects were required to complete the questionnaire in Chinese. The questionnaire comprised scale questions, multiple-choice questions and close-ended questions. Age, occupation, educational level, residence, number of eyes diagnosed with KC, year of first-time RGPCL wear, family history of KC, and corneal CXL surgery history were collected from the demographic questionnaire. The vision-related quality of life questionnaire comprised questions about comfort, visual acuity, overall satisfaction, quality of life, and adaptation to the RGPCL wear.

In order to clarify the validity of the questionnaire, Cronbach's alpha and Kaiser-Meyer-Olkin (KMO) tests were used. The results were 0.78 and 0.87, respectively, which indicated the acceptable reliability and good validity of the questionnaire.

The details of the questionnaire survey are available online at Supplementary File S1: Questionnaire Survey.

### 2.7. Generalised Estimating Equation (GEE)

A logistic-transformed generalised estimating equation (GEE) regression was used to measure the effect of factors on the long-term compliance with RGPCL wear.

The inappropriate analysis of data for one eye or both eyes of the same subjects without accounting for inter-eye correlation could lead to a biased or inefficient estimation of the difference between the two groups. In order to adjust for potential correlations between the two eyes of the same subject, the GEE model was used. The GEE model examines whether independent variables could predict the odds of a particular dichotomous outcome, either for the compliant or non-compliant group. The odds ratios (ORs) and their 95% confidence intervals (CIs) were computed using the available data from each patient. Both single-factor and multiple-factor analyses were performed using GEE.

### 2.8. Statistical Analyses

Demographics and baseline characteristics—such as age, sex, K1, K2, CDVA, and answers to the questionnaire—were summarised by the compliant and non-compliant groups. Differences in characteristics between the groups were tested using the chi-squared, corrected chi-squared, and Fisher’s exact tests for the categorical variables, and one-way analysis of variance for the normally distributed continuous variable.

Two-sided values of *p* < 0.05 were considered statistically significant. All of the statistical analyses were performed using R software (version 4.0.5).

## 3. Results

A total of 208 patients (416 eyes) were reviewed, of which 42 eyes were excluded due to incomplete information on the answer sheet. Finally, 189 patients with KC (374 eyes) were included in the analysis.

### 3.1. Demographic Characteristics

Table 1 presents the respondents’ demographic data. The mean age of the compliant group was 28.9 ± 7.7 years, which was not significantly different from that of the non-compliant group (30.1 ± 7.5 years, *p* = 0.236). Respondents diagnosed with bilateral KC had better compliance with RGPCL wear than those diagnosed with unilateral KC (*p* = 0.0167).

### 3.2. Corneal Topography Measurements, Refractive Results and RGPCL Parameters

Table 2 presents the clinical data of 189 patients with KC (374 eyes). There were no significant differences between the compliant and non-compliant groups in terms of their corneal topography measurements (K1, K2, Kmax, TCT), refractive results (sphere, cylinder, CDVA with spectacles, CDVA with RGPCLs), and RGPCL parameters (base curve, diameter, power) (all *p* > 0.05).

Table 3 presents a comparison of the topographic measurements between the patients with KC who received CXL surgery and those who did not receive CXL surgery. There were no significant differences in K2 and TCT between the two groups (all *p* > 0.05). The K1 and Kmax of the patients who received CXL surgery were significantly higher than those of the patients who did not undergo CXL surgery (49.63 ± 8.12 vs. 47.14 ± 6.98, *p* = 0.003; 61.21 ± 13.24 vs. 57.39 ± 11.63, *p* = 0.007, respectively).

### 3.3. Questionnaire Survey

**Figure 1:**
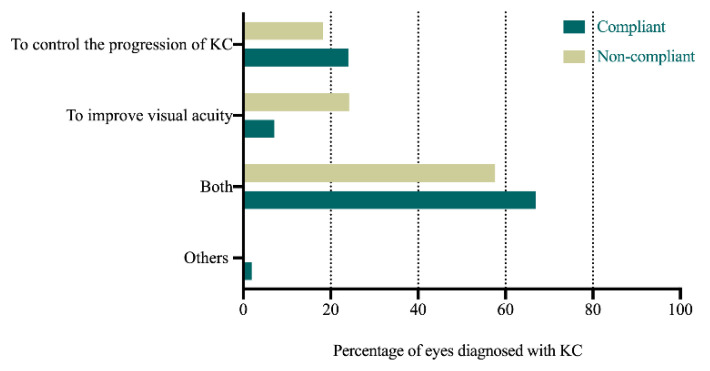
Comparison of the answers to the question “Why did you choose rigid gas-permeable contact lens?” between the compliant and non-compliant groups (KC, keratoconus).

**Figure 2:**
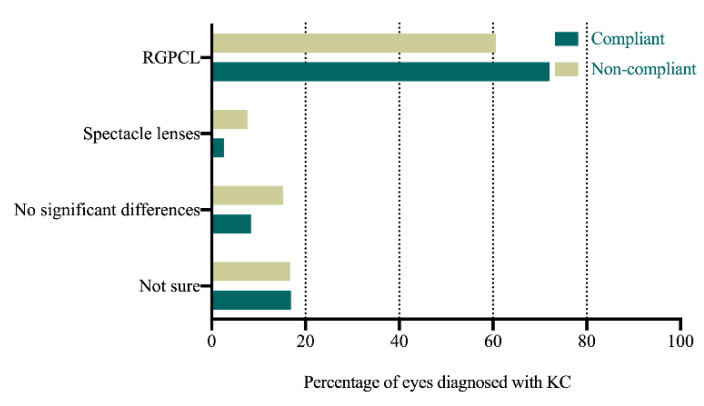
Comparison of the answers to the question “In which treatment do you have better visual acuity?” between the compliant and non-compliant groups (RGPCL, rigid gas-permeable contact lens; KC, keratoconus).

**Figure 3:**
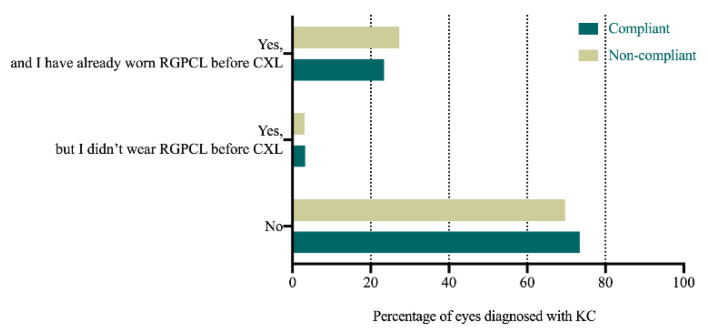
Comparison of the answers to the question “Have you undergone corneal collagen cross-linking surgery?” between the compliant and non-compliant groups (RGPCL, rigid gas-permeable contact lens; CXL, corneal collagen cross-linking; KC, keratoconus).

Table 4 and Figure 1, Figure 2 and Figure 3 present the comparison between the compliant and non-compliant groups in the answers to the questionnaire.

The KC patients’ subjective experience with RGPCL wear—including visual acuity (*p* = 0.006), overall satisfaction (*p* < 0.001), quality of life (*p* < 0.001), and adaptation to RGPCL wear (*p* < 0.001)—significantly influenced their long-term lens-wear compliance. The patients with KC who gained better visual acuity and quality of life with RGPCL wear were more likely to insist on wearing RGPCLs. Similarly, patients with KC who became used to the RGPCL wear in the short term and felt satisfied with lens wear would be more compliant with RGPCL wear. In contrast, patients with KC who were compliant with RGPCL wear were barely affected by the cost (*p* = 0.002) or the short-term discomfort with RGPCLs (*p* < 0.001) (Table 4).

There were no significant differences between the compliant and non-compliant groups in CXL surgery (*p* = 0.798) (Figure 3).

### 3.4. Generalised Estimating Equation (GEE)

A total of 283 eyes of 154 patients with KC with comprehensive clinical data were analysed using GEE.

#### 3.4.1. GEE of the Demographic and Clinical Data

Table 5 presents the results of the GEE analysis of the objective variables. As a result, the objective variables—including corneal tomographic measures (K1, K2, Kmax, TCT), refractive results (sphere, cylinder, CDVA with spectacles, CDVA with RGPCLs), and RGPCL parameters (base curve, diameter, power)—had no effect on long-term compliance with RGPCL wear. Neither the educational level nor resident location of the patients affected their long-term compliance with RGPCL wear (all *p* > 0.05).

#### 3.4.2. GEE of the Answers to the Questionnaire Survey

Table 6 presents the GEE results of the answers to the questionnaire with regard to the subjective questions.

The reason for wearing RGPCLs, overall satisfaction, and quality of life with RGPCL wear had a significant effect on the long-term compliance with RGPCL wear. Patients with KC who attempted both to control the progression of KC and improve visual acuity with RGPCL wear (OR 3.41, 95% CI 1.14–10.19, *p* = 0.03) were more likely to insist on wearing RGPCLs than those who only attempted to improve visual acuity. Patients with KC who agreed (OR 13.89, 95% CI 1.37–140.42, *p* = 0.03) or strongly agreed (OR 19.37, 95% CI 4.94–75.97, *p* < 0.001) with the statement that “I am satisfied with RGPCL wear overall” had better compliance with RGPCL wear than those who disagreed. Patients with KC who agreed (OR 7.12, 95% CI 2.29–22.18, *p* < 0.001) or strongly agreed (OR 9.49, 95% CI 1.71–52.84, *p* = 0.01) with the statement “My quality of life has improved due to the improvement of visual acuity with RGPCL wear” were more likely to insist on wearing RGPCLs than those who were not sure about it.

In the multiple-factor analysis, the quality of life with RGPCL wear had a positive effect on compliance with RGPCL wear. The ORs were 6.5 (95% CI 1.29–32.67, *p* = 0.02) for ‘agree’ and 14.12 (95% CI 2.15–92.84, *p* = 0.01) for ‘strongly agree’ compared with ‘not sure’.

## 4. Discussion

In the current study, patients with bilateral KC were more likely to insist on wearing RGPCLs than those with unilateral KC, which is similar to the result of a previous study [15]. Patients with unilateral KC may dwell on the unaffected eye and still meet daily visual needs. Therefore, they would discontinue RGPCL wear due to having less need of them compared with patients with bilateral KC.

Russell et al. [15] also reported that the further the distance patients live from the hospital, the less likely they are to continue wearing RGPCLs. There was no statistical difference between patients’ resident locations with regard to their compliance with RGPCL wear in the current study. With the improvement of the distribution of medical resources in China, regular follow-up visits after RGPCL treatment can be performed at local hospitals, which largely avoids cross-city travel. Therefore, patients who do not live in Shanghai are still compliant with RGPCL wear.

Unexpectedly, patients with KC with better CDVA wearing RGPCLs, compared to those with spectacles, were not more likely to insist on wearing RGPCLs. Previous studies have revealed that RGPCLs can provide better visual acuity than spectacles for patients with KC [4,5,16]. The current study also found that CDVA significantly improved by more than two lines in both compliant and non-compliant groups. However, the compliance with RGPCLs was not affected by the difference between CDVA with RGPCLs and spectacles. Despite the significant improvement in visual acuity, the subjective feelings were similar between the two groups, suggesting that the subjective measures play a more important role in the long-term compliance with RGPCL wear.

The severity of KC, reflected by the thickness and keratometry of the cornea, had no effect on the compliance of RGPCL wear in the current study. In the CLEK study [17], no significant association was found between patient-reported lens comfort and disease severity, which agrees with the findings of the current study. In contrast, another study [12] suggested that patients with advanced KC showed significantly reduced wearing time (from 10.4 h per day in the mild group and 9.6 h per day in the moderate group to 4.8 h per day in the severe group). The authors proposed that wearing RGPCLs in patients with severe KC could result in discomfort, ocular pain, and foreign body sensation. The discrepancy among the above-mentioned studies may be because all of the patients recruited in Wu et al.’s study used RGPCLs for the first time, while patients in both the current study and the CLEK study had already worn RGPCLs for a significant period before the questionnaire survey. The patients in the current study might have become used to the discomfort induced by RGPCL wear, even if the severity of the disease had progressed and the cornea steepened during the extended wear of the lens. In addition, the inclusion criteria associated with corneal shape varied among the above-mentioned three studies. The K2 in Wu et al.’s study covered a broad range, from <45 D to >52 D (Tomey KC screening system), and the patients were classified into mild (K2 < 45 D), moderate (45 D ≤ K2 ≤ 52 D), and severe (K2 > 52 D) groups according to their K2. In contrast, 95.4% of patients had a K2 of at least 45 D (TMS-1, EyeSys, Visioptic EH270, Humphrey MasterVue) in the CLEK study [18,19], and the Kmax (Pentacam HR imaging system) averaged 58.79 ± 12.04 D and 56.76 ± 12.83 D for the two groups in the current study, with the overall corneal shape being steeper and covering a relatively small range of keratometry. Therefore, the ability to test the differences in subjective feeling among these patients was compromised.

The current study also found that CXL surgery did not increase compliance with RGPCL wear. Previous studies have shown that RGPCL wear post-CXL was relatively well tolerated due to the decreased corneal sensitivity and the flattening effect of CXL [11,20,21]. Unlu et al. [11] also found that the mean duration of RGPCL wear increased after CXL surgery. In the current study, the vast majority of patients with KC (87.8%) from the compliant group were already wearing RGPCLs before CXL. Therefore, CXL surgery would not improve the long-term compliance with RGPCL wear to a greater extent.

Patient comfort and satisfaction while wearing RGPCLs are important factors that affect patient compliance. A previous study reported that patients with KC would decrease lens-wearing time or stop wearing RGPCLs due to uncomfortable feelings [12]. In the current study, discomfort with RGPCL wear did not negate the need for treatment in the compliant group. Patients with KC attached more importance to visual acuity than discomfort, which could be tolerated. In addition, the compliance with RGPCL wear was not affected by the high cost of RGPCL treatment, which was different from the result of another study conducted in Jordan [22]. Bakkar et al. [22] revealed that the high cost of RGPCLs and the lack of governmental health insurance or subsidised medical services which cover the lens cost are important barriers for patients with KC to the wearing of RGPCLs. Patients who were unemployed and had low income were more likely to report the cost of the lenses as a decisive barrier to use. They were less likely to repeatedly change the RGPCLs because of the progression of KC. Patients who were not working and patients with low monthly income accounted for 47.5% and 83.8%, respectively, in their study. In contrast, the ratio of non-working patients in the current study was 27.0%, and the occupation in the majority of the working patients was stable, decent, and well paid. Therefore, the high cost of RGPCLs was not an important factor for the long-term compliance of RGPCL wear in the current study.

Furthermore, the reason for choosing RGPCLs for the refractive treatment of KC is another important factor for the compliance with RGPCL wear. Patients who attempt to control KC progression with RGPCLs are more likely to continue wearing RGPCLs. Before the consensus of KC treatment was reached in 2019 [23], some doctors assumed that RGPCLs would have an effect in controlling the progression of the disease, and prescribed the lenses for this purpose. Patients chose to wear RGPCLs at the doctors’ recommendation, and there was a positive expectation of long-term RGPCL wear. Araki et al. [4] revealed that the effects of long-term RGPCL wear had no effect on KC progression based on corneal tomographic evaluation over 5–6 years. Despite being a positive initiative, patients with KC should be fully informed before RGPCL fitting in order to address their unrealistic expectations in clinical practice.

This study has some limitations. First, the sample sizes between the compliant group (66 eyes) and the non-compliant group (308 eyes) was significantly different, which may have led to bias in the results. Second, the classification of bilateral versus unilateral KC was based on the topographic findings before RGPCL wear and on the answers provided by patients per se. Some of the originally ‘unilateral’ patients might have progressed to ‘bilateral’ at the time of the survey, thereby confounding the comparison between the two groups.

## 5. Conclusions

The current study revealed that patients with bilateral KC are more compliant with RGPCL wear than those with unilateral KC in the long-term. Ocular biometrics—including corneal topographic measurements, refractive results, and RGPCL parameters—did not affect the long-term compliance with RGPCL wear. Patients with KC who attempt to control KC progression with RGPCLs, who gain better visual acuity and quality of life with RGPCL wear, who become used to the RGPCLs in the short term, and who feel more satisfied with lens wear are more likely to insist on wearing RGPCLs. Improved visual acuity with RGPCLs compared with spectacles and CXL surgery history did not have a positive effect on compliance with long-term RGPCL wear. Short-term discomfort with RGPCL wear, a long distance from resident locations to the hospital, and the high cost of RGPCLs have no negative effect on long-term compliance with lens wear.

## Figures and Tables

**Table 1:** Demographics of the respondents (n = 189).

Variable	Frequency (%)
Sex	
Female	67 (35.4)
Male	122 (64.6)
Educational level	
High school education or less	41 (21.7)
College education or above	148 (78.3)
Occupation	
Working	138 (73.0)
Not working	51 (27.0)
Residence	
Local	74 (39.1)
Non-local	115 (60.9)
Family history with KC	
Yes	4 (2.1)
No	185 (97.9)
Eyes diagnosed with KC	
Unilateral	52 (27.5)
Bilateral	137 (72.5)
Compliance of RGPCL wear	
Yes	155 (82.0)
No	34 (18.0)
CXL surgery history	
Yes	51 (27.0)
No	138 (73.0)

KC, keratoconus; RGPCL, rigid gas-permeable contact lens; CXL, corneal collagen cross-linking.

**Table 2:** Clinical data of the respondents (308 eyes, compliant; 66 eyes, non-compliant).

	Compliant (Mean ± SD)	Non-Compliant (Mean ± SD)	*p* Value
K1 (D)	48.11 ± 7.40	46.46 ± 7.22	0.236
K2 (D)	51.77 ± 8.52	50.63 ± 8.37	0.099
Kmax (D)	58.79 ± 12.04	56.76 ± 12.83	0.219
TCT (μm)	461.2 ± 56.9	465.3 ± 71.4	0.617
Spherical error (D)	−4.50 ± 4.53	−5.05 ± 5.19	0.391
Cylindrical error (D)	−3.12 ± 2.67	−3.51 ± 3.02	0.301
Spectacle CDVA (logMAR)	0.28 ± 0.49	0.24 ± 0.47	0.190
RGPCL base curve (mm)	6.79 ± 0.86	6.98 ± 0.93	0.114
RGPCL diameter (mm)	8.94 ± 0.48	8.95 ± 0.45	0.850
RGPCL power (D)	−10.94 ± 7.12	−10.09 ± 7.94	0.405
RGPCL CDVA (logMAR)	0.08 ± 0.70	0.08 ± 0.64	0.554

K1, flat keratometry; K2, steep keratometry; Kmax, maximum keratometry; TCT, thinnest corneal thickness; RGPCL, rigid gas-permeable contact lens; CDVA, corrected distance visual acuity.

**Table 3:** Topographic measurements compared between the patients with and without CXL history (102 eyes, with CXL history; 272 eyes, without CXL history).

	with CXL History (Mean ± SD)	without CXL History (Mean ± SD)	*p* Value
K1 (D)	49.63 ± 8.12	47.14 ± 6.98	0.003 *
K2 (D)	52.79 ± 10.18	51.11 ± 7.74	0.089
Kmax (D)	61.21 ± 13.24	57.39 ± 11.63	0.007 *
TCT (μm)	453.1 ± 55.7	465.2 ± 60.8	0.079

K1, flat keratometry; K2, steep keratometry; Kmax, maximum keratometry; TCT, thinnest corneal thickness. * *p* < 0.05.

**Table 4:** Comparison of different answers to the questionnaire survey between the compliant and non-compliant groups (308 eyes, compliant; 66 eyes, non-compliant) (%).

Do You Agree with the Statement		Strongly Agree	Agree	Not Sure	Disagree	Strongly Disagree	*p* Value
1 ‘I will give up RGPCL because of its high cost’?	Compliant Non-compliant	1.9 3.0	4.5 13.7	27.0 33.3	42.9 43.9	23.7 6.1	0.002 *
2 ‘My visual acuity improved with RGPCL wear’?	Compliant Non-compliant	54.5 59.1	26.9 9.1	14.6 22.7	3.3 9.1	0.7 0.0	0.006 *
3 ‘I am satisfied with RGPCL wear overall’?	Compliant Non-compliant	8.1 3.1	78.3 21.9	6.8 32.8	6.2 29.7	0.6 12.5	<0.001 *
4 ‘I am worried about the decentration or loss of RGPCL when I am wearing RGPCL’?	Compliant Non-compliant	36.5 53.0	50.7 40.9	5.6 6.1	4.6 0.0	2.6 0.0	0.049 *
5 ‘My quality of life has improved due to the improvement of visual acuity with RGPCL wear’?	Compliant Non-compliant	19.0 6.3	70.9 34.4	8.8 25.0	1.3 28.1	0.0 6.2	<0.001 *
6 ‘I got used to the RGPCL within 2 weeks after the first commencement of lens wear’?	Compliant Non-compliant	12.0 3.0	65.9 33.3	8.1 10.6	12.0 36.4	2.0 16.7	<0.001 *
7 ‘Discomfort with RGPCL wear has negatively affected my need for it’?	Compliant Non-compliant	8.8 45.4	39.3 39.4	14.3 9.1	33.7 6.1	3.9 0.0	<0.001 *
8 ‘I often experience discomfort such as eye redness or eye pain with RGPCL wear’?	Compliant Non-compliant	9.7 10.6	37.3 44.0	21.1 30.3	28.6 12.1	3.3 3.0	0.077
9 ‘I am afraid that long-term wear of RGPCL will have side effects on my eyes’?	Compliant Non-compliant	11.2 3.1	28.9 20.3	35.2 48.4	23.4 21.9	1.3 6.3	0.01 *

RGPCL, rigid gas-permeable contact lens. * *p* < 0.05.

**Table 5:** Generalised estimating equation of the demographic and clinical data of the respondents.

Variables		Single-Factor Analysis		Multiple-Factor Analysis	
		Odds Ratio (95% CI)	*p* Value	Odds Ratio (95% CI )	*p* Value
Sex	Male Female	Referent 1.04 (0.43–2.50)	Referent 0.93		
Age		0.99 (0.95–1.05)	0.82		
Educational level	College or above Primary school Middle school High school (n = 21)	Referent 0.53 (0.10–2.92) 0.90 (0.28–2.95)	Referent 0.47 0.87		
Residence	Non-local Local	Referent 0.95 (0.41–2.17)	Referent 0.90	Referent 2.27 (0.75–6.86)	Referent 0.15
K1		1.00 (0.99–1.01)	0.47	1.23 (0.90–1.67)	0.19
K2		1.00 (0.99–1.01)	0.98	0.81 (0.67–0.99)	0.04 *
Kmax		1.00 (1.00–1.01)	0.93	1.04 (0.96–1.11)	0.34
TCT		1.00 (1.00–1.00)	0.85	1.01 (0.98–1.05)	0.51
Spherical error		1.00 (0.98–1.03)	0.73	0.93 (0.83–1.04)	0.22
Cylindrical error		1.00 (0.98–1.03)	0.77	1.09 (0.89–1.34)	0.41
Spectacle CDVA (logMAR)		0.93(0.82–1.06)	0.30	0.36 (0.04–3.35)	0.37
RGPCL base curve		0.97 (0.90– 1.03)	0.30		
RGPCL diameter		0.98 (0.83–1.16)	0.81		
RGPCL power		1.00 (0.99–1.01)	0.70	0.94 (0.82–1.08)	0.39
RGPCL CDVA (logMAR)		1.02 (0.71–1.47)	0.93	1.96 (0.27–14.32)	0.51

K1, flat keratometry; K2, steep keratometry; Kmax, maximum keratometry; TCT, thinnest corneal thickness; CDVA, corrected distance visual acuity; RGPCL, rigid gas-permeable contact lens; CI, confidence interval. * *p* < 0.05.

**Table 6:** Generalised estimating equation results of the answers to the questionnaire survey from the respondents.

Do You Agree with the Statement		Single-Factor Analysis		Multiple-Factor Analysis	
		Odds Ratio (95% CI)	*p* Value	Odds Ratio (95% CI)	*p* Value
1 ‘I will give up RGPCL because of its high cost’?	Disagree	Referent	Referent		
	Strongly disagree	0.67 (0.27–1.69)	0.40		
	Not sure	6.09 (0.75–49.16)	0.09		
	Agree	0.42 (0.04–5.04)	0.50		
	Strongly agree	0.29 (0.06–1.45)	0.13		
2 ‘My visual acuity improved with RGPCL wear’?	Disagree	Referent	Referent		
	Strongly disagree	0.50 (0.07–3.63)	0.49		
	Agree	4.15 (0.96–17.88)	0.06		
	Strongly agree	1.44 (0.52–3.99)	0.48		
	Not sure (n = 27)				
3 ‘I am satisfied with RGPCL wear overall’?	Disagree	Referent	Referent		
	Strongly disagree	0.74 (0.18–3.15)	0.69		
	Not sure	0.39 (0.03–4.78)	0.46		
	Agree	13.89 (1.37–140.42)	0.03 *		
	Strongly agree	19.37 (4.94–75.97)	<0.001 *		
4 ‘I am worried about the decentration or loss of RGPCL when I am wearing RGPCL’?	Not sure	Referent	Referent		
	Strongly disagree	0.34 (0.04–2.95)	0.32		
	Strongly agree	0.95 (0.11–8.58)	0.97		
	Agree (n = 79)	4.30 × 10^17^ (4.26 × 10^17^–4.35 × 10^17^)	<0.001 *		
	Disagree (n = 5)				
5 ‘My quality of life has improved due to the improvement of visual acuity with RGPCL wear’?	Not sure	Referent	Referent	Referent	Referent
	Disagree	0.10 (0.01–0.96)	0.05	0.14 (0.02–0.99)	0.05
	Agree	7.12 (2.29–22.18)	<0.01 *	6.5 (1.29–32.67)	0.02 *
	Strongly agree	9.49 (1.71–52.84)	0.01 *	14.12 (2.15–92.84)	0.01 *
	Strongly disagree (n = 0)				
6 ‘I got used to the RGPCL within 2 weeks after the first commencement of lens wear’?	Disagree	Referent	Referent		
	Strongly disagree	1.60 (0.38–6.74)	0.52		
	Not sure	0.28 (0.04–1.86)	0.19		
	Strongly agree	8.96 (0.99–81.06)	0.05		
	Agree	4.29 (1.47–12.53)	0.01 *		
7 ‘Discomfort with RGPCL wear has negatively affected my need for it’?	Not sure	Referent	Referent	Referent	Referent
	Disagree	7.54 (0.73–77.66)	0.09	5.89 (0.74–46.73)	0.09
	Agree	0.90 (0.22–3.66)	0.88	0.46 (0.08–2.49)	0.37
	Strongly agree	0.11 (0.03–0.50)	<0.001 *	1.91 (0.35–10.41)	0.45
	Strongly disagree (n = 6)				
8 ‘I often experience discomfort such as eye redness or eye pain with RGPCL wear’?	Disagree	Referent	Referent		
	Strongly disagree	0.31 (0.09–1.16)	0.08		
	Not sure	0.50 (0.05–5.38)	0.57		
	Strongly agree	0.27 (0.06–1.27)	0.10		
	Agree	0.41 (0.12–1.40)	0.16		
9 ‘I am afraid that long-term wear of RGPCL will have side effects on my eyes’?	Disagree	Referent	Referent		
	Strongly disagree	0.60 (0.21–1.70)	0.34		
	Not sure	0.41 (0.03–5.30)	0.50		
	Strongly agree	2.85 (0.31–26.03)	0.35		
	Agree	1.81 (0.47–7.03)	0.39		

RGPCL, rigid gas-permeable contact lens; CI, confidence interval. * *p* < 0.05.

## Data Availability

The data presented in this study are available on request from the corresponding author.

## Supplementary Materials

The following supporting information can be downloaded at: https://www.mdpi.com/article/10.3390/jcm11041091/s1, Supplementary File S1: Questionnaire survey.
