# Supplementary material for: Factors Affecting Long-Term Compliance with Rigid Gas-Permeable Contact Lens Wear in Patients with Keratoconus
Source: J Clin Med. 2022 Feb 18;11(4):1091. doi: 10.3390/jcm11041091 (PMC8878038; doi:10.3390/jcm11041091)
Supplement: Supplementary file 1 [file jcm-11-01091-s001.zip › jcm-1510600-supplementary.pdf]

Supplementary File S1

Questionnaire survey.

1. What is your name?__________

2. What is your date of birth?__________

3. What is your sex? __________

4. What is the city you are living in? __________

5. What is your occupation? Please choose between:

   □ Auditor

   □ Consultant

   □ Management personnel

   □ Administrative/Support staff

   □ Technology/ Research staff

   □ Teacher

   □ Full-time student

   □ HR staff

   □ Production personnel

   □ Marketing personnel/public relation practitioner

   □ Civil personnel/ Clerk

   □ Sales personnel

   □ Professional(accountant, lawyer, architect, medical staff, journalist)

   □ Others __________

6. What is your educational level?

   □ Primary school

   □ Middle school

   □ High school

 College or above

7. When were you first diagnosed with keratoconus? __________

8. Which eyes are you diagnosed with keratoconus?

 The right eye

 The left eye

 Bilateral eyes

9. When did you first wear rigid gas permeable contact lens (RGPCL)? __________

10. Are you still wearing your RGPCL?

 Yes

 No

11. If the answer is NO, pleaser answer:

1) When did you stop wearing RGPCL? __________

2) Why did you stop wearing RGPCL? __________

12. Do you have family members who were also diagnosed keratoconus?

 Yes

 No

13. If the answer is Yes, please answer:

1) How many family members were diagnosed keratoconus? __________

2) Relative 1: What is the relationship between you and her/him? __________

What is the age of your relative? __________

What is the gender of your relative? __________

3) Relative 2: What is the relationship between you and her/him? __________

What is the age of your relative? __________

What is the gender of your relative? __________

4) …

14. Why did you choose RGPCL?

 To control the progression of KC

 To improve visual acuity

 Both

15. Do you agree with the statement that “I will give up RGPCL because of its high cost”?

 Strongly disagree

 Disagree

 Not sure

 Agree

 Strongly agree

16. Do you agree with the statement that “My visual acuity improved with RGPCL wear.”?

 Strongly disagree

 Disagree

 Not sure

 Agree

 Strongly agree

17. Do you agree with the statement that “I am satisfied with RGPCL wear overall.”?

 Strongly disagree

 Disagree

 Not sure

 Agree

 Strongly agree

18. Do you agree with the statement that “I am worried about the decentration or loss of

RGPCL when I am wearing RGPCL.”?

 Strongly disagree

 Disagree

 Not sure

 Agree

 Strongly agree

19. Do you agree with the statement that “My quality of life has improved due to the improvement of visual acuity with RGPCL wear”?

 Strongly disagree

 Disagree

 Not sure

 Agree

 Strongly agree

20. Do you agree with the statement that “I got used to the RGPCL within 2 weeks after the first commencement of lens wear”?

 Strongly disagree

 Disagree

 Not sure

 Agree

 Strongly agree

21. Do you agree with the statement that “Discomfort with RGPCL wear has negatively affected my need for it.”?

 Strongly disagree

 Disagree

 Not sure

 Agree

 Strongly agree

22. Do you agree with the statement that “I often experience discomfort such as eye redness or eye pain with RGPCL wear.”?

 Strongly disagree

 Disagree

 Not sure

 Agree

 Strongly agree

23. Do you agree with the statement that “I am afraid that long-term wear of RGPCL will have side effects on my eyes. ”?

 Strongly disagree

 Disagree

 Not sure

 Agree

 Strongly agree

24. In which treatment do you have better visual acuity?

 RGPCL

 Spectacle lenses

 No significant differences

 Not sure

25. Have you undergone corneal collagen cross-linking(CXL) surgery?

 No.

 Yes, but I didn’t wear RGPCL before CXL.

 Yes, and I have already worn RGPCL before CXL.
